# Does reduced smoking if you can’t stop make any difference?

**DOI:** 10.1186/s12916-015-0505-2

**Published:** 2015-10-12

**Authors:** Rachna Begh, Nicola Lindson-Hawley, Paul Aveyard

**Affiliations:** UK Centre for Tobacco and Alcohol Studies, Nuffield Department of Primary Care Health Sciences, University of Oxford, Oxford, UK

**Keywords:** Harm reduction, Nicotine, Smoking

## Abstract

**Background:**

Promoting and supporting smoking reduction in smokers with no immediate intention of stopping smoking is controversial given existing fears that this will deter cessation and that reduction itself may not improve health outcomes.

**Discussion:**

Evidence shows that smokers who reduce the number of daily cigarettes smoked are more likely to attempt and actually achieve smoking cessation. Further, clinical trials have shown that nicotine replacement therapy benefits both reduction and cessation. Worldwide data suggests that ‘non-medical’ nicotine is more attractive to people who smoke, with electronic cigarettes now being widely used. Nevertheless, only one small trial has examined the use of electronic cigarettes to promote reduction, with direct evidence remaining inconclusive. It has been suggested that long-term reduced smoking may directly benefit health, although the benefits are small compared with cessation.

**Summary:**

The combined data imply that smoking reduction is a promising intervention, particularly when supported by clean nicotine; however, the benefits are only observed when it leads to permanent cessation.

## Background

Smoking reduction, defined herein as a decrease in the number of cigarettes smoked per day, is a strategy used by smokers to moderate the health and financial effects of smoking and ease towards complete cessation [1]. It is advocated by some health professionals for smokers who have no immediate intention of quitting or those who have been unable to do so. Despite approximately half of all smokers in England currently attempting a reduction in smoking [2], this strategy remains controversial. Concerns have been raised regarding smoking reduction being seen as a favourable alternative compared to complete cessation, thus possibly decreasing the likelihood of future cessation [3]. Nevertheless, eventual cessation success may be influenced by the methods used to reduce smoking, particularly given the several options currently available to support this, including pharmacotherapies, such as nicotine replacement therapy (NRT) or varenicline, oral tobacco products including snus, and more recently, novel devices such as electronic cigarettes (e-cigarettes). However, if smokers are unable to quit following reduction, it is important to understand whether this affects their health outcomes in order to inform the current debate.

In this opinion piece, the main issues related to smoking reduction and cessation are considered and evaluated based on the available evidence. The discussion includes an evaluation of whether promoting smoking reduction directly or indirectly deters cessation, a summary of the methods available to reduce smoking, and discussion of evidence that reduction itself may decrease the harm from smoking even if cessation is not achieved.

### Reduction may lead to complete cessation

Several theoretical factors indicate that smoking reduction promotes rather than deters cessation. Firstly, smoking reduction may be a more attainable goal compared to complete cessation and is more desirable than regular smoking; additionally, once achieved, it may encourage further efforts to achieve cessation (Michie S, Personal communication). Further, regular smoking results in neuroadaptation, which manifests in the features recognised as tobacco addiction [4]; smoking reduction could reverse these adaptations and decrease the severity of withdrawal and cravings when smokers abstain altogether. Indeed, withdrawal effects and cravings are the main barriers to achieving cessation and contributing to relapse [5]. The neural processes induced by cigarette smoking lead to the formation of conditioned relationships between environmental stimuli and smoking. A reduction in smoking may disrupt these relationships so that a desire to smoke is less likely to be triggered by previous cues to do so [4]. Another hypothesis concerns ‘shaping’, which involves making successive, positively reinforced approximations of a target behaviour, thus encouraging the desired final behaviour [6]. In the context of smoking, gradually reducing the number of daily cigarettes may induce intermittent reinforcement, offering encouragement to and increasing the likelihood of quitting altogether. Finally, the positive reinforcement that smokers may experience when gradually reducing the number of cigarettes smoked could also increase their self-efficacy – an individual’s belief in their ability to succeed. Increases in self-efficacy are thought to heighten the likelihood that a final goal – in this case cessation – will be achieved [7].

Despite all of the above factors being merely hypothetical, there is evidence of an association between smoking reduction and subsequent cessation. A telephone survey of 1,000 daily smokers in the US suggested that most of those who reduced their smoking did so as a stepping stone to cessation [8]. A qualitative systematic review, including 19 observational studies (smokers who had spontaneously reduced smoking or not) or randomised controlled trials (RCTs; smokers instructed to reduce smoking or not) reporting on changes in the number of cigarettes smoked per day and future cessation, showed no indication that reduction adversely affected the rate of future quitting [9]. Indeed, 16 of the included studies indicated that reduction was associated with higher rates of eventual cessation. These findings are supported by a later review of 10 RCTs, in which all of the included trials compared either a pharmacological, behavioural, or combined smoking reduction intervention in smokers not yet ready to quit to at least one control group, defined as placebo, no treatment, or minimal psychological intervention [10]. Meta-analysis of the pharmacological and combined interventions showed that both reduction interventions increased the likelihood of long-term abstinence (6 months or over). Nevertheless, there was insufficient evidence available to reach a conclusion on whether behavioural support for reduction alone enhanced future cessation. A key methodological issue with these reviews is that most reduction studies only report 7-day, point-prevalence smoking abstinence at the end of follow-up, which will overestimate long-term cessation. Future studies should follow published recommendations for measuring abstinence in trials of smokers who are not ready to quit [11].

### Supporting smoking reduction and subsequent cessation

The aim of smoking reduction is to decrease the exposure to tobacco toxins and facilitate subsequent cessation. However, evidence suggests that smokers involuntarily undermine their best intentions by compensating, namely adjusting the way they smoke, for example, by taking longer, deeper puffs to maintain the same levels of nicotine [12]. Switching to alternative, less hazardous sources of nicotine while smoking fewer cigarettes may limit the extent of compensation and perhaps reduce the harm of smoking.

Results from several RCTs have demonstrated that smokers who are unmotivated to quit are more likely to reduce their cigarette consumption when using NRT compared with placebo [13]. Many countries have licenced the use of NRT for the purpose of reducing smoking and eventually quitting. NRT is intermittently promoted for this use, with the UK promoting harm reduction as national policy, but is not implemented in the UK as it is in RCTs, where participants have regular support and supervision. UK population data suggests that using NRT to reduce smoking is not associated with lower cigarette consumption relative to reduction without NRT [14]. However, it is associated with increased motivation to quit and higher cessation rates [15, 16].

Results from other countries suggest that non-medical forms of nicotine may be more popular. Smokeless tobacco is another non-combustible source of nicotine. Although it is more hazardous than NRT, some oral tobacco products, such as Swedish snus, have been estimated to be approximately 90 % less harmful than smoking [17]. In Sweden, a reduction in smoking among men has been attributed to substituting cigarettes with snus, leading to lower tobacco-related mortality rates than in other European countries [18]. Cross-sectional surveys show that snus use is associated with smoking reduction and a higher probability of cessation [19–21]. Some RCTs report on the potential benefits of smokeless tobacco, a non-combustible source of nicotine, on increasing cessation rates in motivated smokers [22, 23], but there are only few comparable trials in smokers who have no intention of quitting. In one pilot study, 31 non-motivated smokers were randomised to receive smokeless tobacco lozenges or to continue smoking cigarettes [24]. Smokeless tobacco use led to significant reductions in smoking and significant increases in two measures of readiness to quit, while no such changes were found in those randomised to continue smoking. Similarly, another pilot study found that those randomised to receive snus reported a reduction in the number of cigarettes smoked per day and greater intention to quit smoking compared with a no-supply control group [25]. However, these findings are preliminary and larger studies are needed to examine whether these interventions do indeed increase cessation rates.

The use of e-cigarettes by regular smokers has been increasing worldwide [26, 27]. Nevertheless, evidence indicating that they are effective in encouraging and supporting smoking reduction and cessation is limited. Despite these being termed cigarettes, they do not contain tobacco and there is no combustion involved; the nicotine included in e-cigarettes is extracted from tobacco and toxicological testing of the vapour reveals high traces of tobacco toxins, exceeding those in NRT, but at concentrations much lower than those in conventional cigarettes [28]. A report commissioned by the World Health Organization [29] and a subsequent review [30] concluded that the health benefits and efficacy of e-cigarettes for harm reduction and cessation were unsupported by the evidence to date, although this has been contested [31]. Data from clinical studies show no adverse effects of their short-term use on cardiovascular function [32]. More trials and studies are needed, particularly with regards to long-term risks. Survey data show that e-cigarettes are most commonly used in the reduction and cessation of cigarette consumption, to alleviate tobacco withdrawal symptoms, and to reduce the harm associated with smoking [33, 34]. Findings from a recent Cochrane review of two RCTs suggest that e-cigarettes with nicotine help smokers to reduce their cigarette consumption and quit compared to placebo [35]. One of the included studies examined the effects of e-cigarette use in smokers not intending to quit, comparing two different e-cigarette nicotine doses with placebo e-cigarettes [36]. The authors found that, at 1 year, more smokers in the nicotine e-cigarette groups were able to reduce their cigarette consumption by at least half and quit compared with the placebo group, though these differences were not statistically significant. Despite the fact that the study was underpowered to detect differences in cessation between groups and used products with low nicotine delivery, the data suggests that e-cigarettes may help those unable to quit to reduce smoking and eventually do so. This is also supported by the findings of prospective cohort studies [37–40].

The effectiveness of e-cigarettes in promoting smoking cessation in current smokers may also depend on the type of e-cigarettes used and the frequency of use [41, 42]. A recent survey found that, compared with non-use, daily use of e-cigarettes while smoking was associated with increased cessation attempts and reduced smoking, but not with final cessation [41]. Hitchman et al. [42] found that daily users of the tank model of e-cigarettes were more likely to have quit at the 1-year follow-up compared with those who reported no use, while non-daily users of e-cigarettes that mimic cigarettes, cigalikes, were less likely to have quit. It is clear that e-cigarette devices are evolving rapidly, gradually becoming more effective in promoting smoking reduction and cessation.

### Can smoking reduction without cessation benefit health?

The prime reason for smokers to reduce or quit smoking is to mitigate the health harms [43]. Smoking causes three main fatal conditions – ischaemic heart disease, lung cancer, and chronic obstructive pulmonary disease (COPD). For each of these conditions, there is a clear dose–response relationship between smoking level and risk of developing or dying from the disease [44–46]. It stands to reason that reduced smoking would mitigate some of the harms, particularly for COPD and lung cancer, where the dose–response relationship is steep. There is epidemiological evidence that supports this reasoning to some extent. In 2007, a systematic review indicated that only one study reported no reduction in the risk of myocardial infarction over 15 years, following a reduction of at least one-half of the initial cigarette consumption [47]. A subsequent study showed evidence that smoking reduction decreased cardiovascular disease rates, reporting a hazard ratio (HR) of reducers over maintainers of 0.77 (95 % confidence interval (CI), 0.66–0.94) [48]. Similarly, another large cohort study produced data that suggested, but did not conclusively show, a reduced risk of infarction and stroke in reducers [49]. A study examining outcomes in peripheral vascular disease showed a reduced risk of progression [47]. Likewise, studies with short follow-up generally showed an improvement in cardiovascular risk factors in response to reduced smoking [47]. Nevertheless, a combined analyses of two Scottish cohorts found no decreased mortality risk for reducers (HR, 0.98; 95 % CI, 0.85–1.13) [50].

There is also mixed evidence on whether smoking reduction decreases the risk of lung cancer. A systematic review found three papers, of which the largest study found a reduction in the risk of lung cancer in reducers compared with maintainers [47]. A subsequent study found no evidence of a reduction (HR, 0.91; 95 % CI, 0.70–1.19) [50]. There is convincing evidence that short-term reductions do decrease the levels of various biological markers of carcinogen exposure or cancer-related changes [47], but the significance for reduced risk is unclear.

Only one study has examined the risk of admission to hospital with COPD exacerbation in relation to reduced exposure and found an HR of 0.93 (95 % CI, 0.73–1.18) [51]. There is clear evidence indicating that reduced smoking decreases COPD and asthma symptoms but not lung function [47].

The interpretation of epidemiological data related to the health benefits of smoking reduction is complex due to three key issues. Firstly, most epidemiological studies measure smoking on two occasions only but make the implicit assumption that smoking reduction measured at the second occasion has been maintained for the entire duration of follow-up. This is problematic since it is clear that smokers experience several phases of trying to reduce then reverting and therefore this reduced consumption may not have been maintained for long. Indeed, data indicates that, from smokers seeking to reduce, approximately 10 % can maintain a reduced consumption for 2 years [52]. Secondly, few studies have used biomarkers of exposure. It is therefore unclear whether those who reduce their smoking frequency and partially compensate by smoking each cigarette more intensively go on to adjust in the long-term [52]. In these cases, the health benefits of reduction would not be as great as might be predicted from the known dose-response relation. Thirdly, all of these studies have included smokers who have reduced without the use of concurrent nicotine. Laboratory data show that, even at high doses, NRT with concomitant smoking has fewer acute effects on biomarkers of cardiovascular risk compared with smoking alone in the short term [53]. The only available long-term studies assessing the health consequences of concurrent use of nicotine and smoked tobacco concern snus; a systematic review of 21 relevant studies found that, in most, the evidence suggested that dual use was associated with reduced risks of all smoking-related conditions compared with smoking alone [54]. However, these studies were not formally statistically combined and, since the benefits were modest and studies small, few risks were individually statistically significant.

## Summary

Smokers who are unable or unwilling to quit would benefit from a gradual reduction in the number of daily cigarettes smoked given its benefit in the likelihood of achieving cessation. Combining reduction attempts with clean nicotine leads to greater reduction and cessation rates. Indeed, e-cigarettes show particular promise since they appeal to smokers and may help them achieve cessation. Nevertheless, their rapid evolution, with newer models outpacing older devices tested in currently published efficacy trials, presents a challenge for researchers and clinicians. Finally, evidence that reduced smoking decreases the harm from smoking is suggestive, but not conclusive. The lack of detailed characterisation of the extent and length of smoking reduction in relation to health outcomes makes it difficult to determine the health benefits. Given the uncertainty of this evidence and the clear benefits of total smoking cessation, the focus in harm reduction should remain on promoting cessation through reduction rather than on reduction as an end goal.

## Abbreviations

CI: Confidence intervals
COPD: Chronic obstructive pulmonary disease
E-cigarette: Electronic cigarette
HR: Hazard ratio
NRT: Nicotine replacement therapy
RCT: Randomised controlled trial
